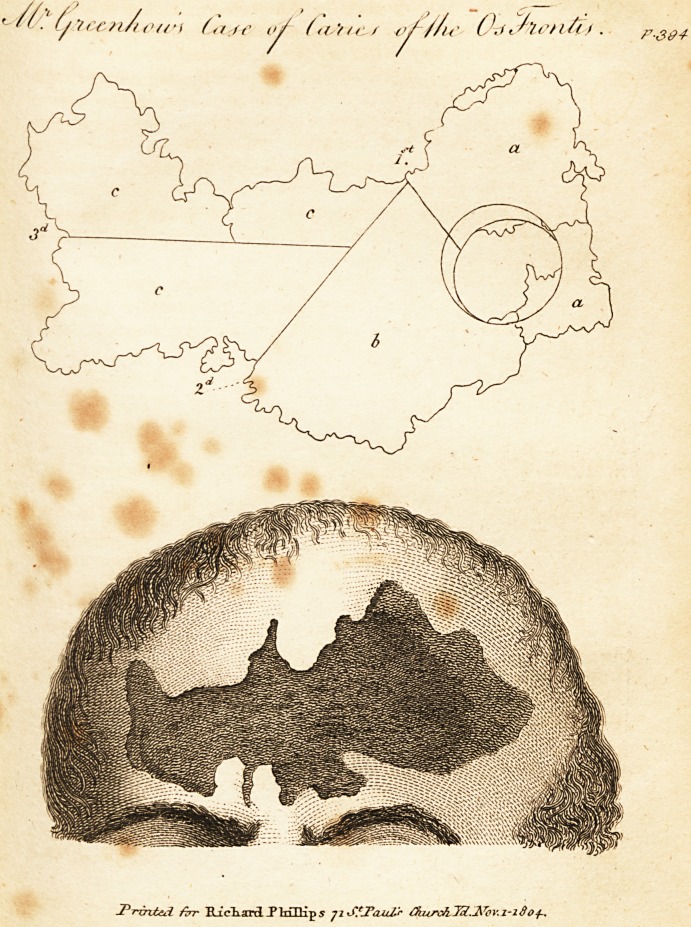# Mr. Greenhow's Case of Caries of the Os Frontis

**Published:** 1804-11-01

**Authors:** E. M. Greenhow

**Affiliations:** North Shields


### *To the Editors of the Medical and Physical Journal.*

GENTLEMEN,

HAVING on a recent occasion found the saw recommended by Mr. Hey of Leeds peculiarly useful, I beg leave to transmit to you a short statement of the case, and a drawing illustrative of it.

I am, &c.

*North Shields, Sept.* 7, 1804. E. M. GREENHOW.

My

My patient is a sailor of about thirty years of age, who from syphilis had an extensive caries of the os frontis.

I shall pass over the medical treatment, as my intention is merely to prove, *in this case*, the superiority of the above instrument over any other we are possessed of.

He became a patient at the Dispensary here in March 1803, at which time it was thought proper to make a perforation with a trephine, to give exit to any pus that might be accumulated between the cranium and dura mater. This was accordingly done by Mr. Burnet, under whose care he then was.

We found, that the bone was completely diseased throughout, and that the dura mater was thickly covered with healthy granulations.

The diseased bone was denuded, at that time, to the extent of five inches in length, and nearly three inches in breadth in one place, by the ulcerative process, and the scalp was detached to a much greater extent. So that it was deemed impossible at that time to remove it.

Finding at length, as his general health improved, that nature was exerting herself to throw off the diseased bone by a gradual dissolution at its connection with the sound part, it was thought right to assist her in her process by removing at least a part of it.

It had occurred to me, that I should find Mr. Hey's saw useful, when it was found proper to remove the bone. I therefore now sawed across from where the perforation had been made by the trephine, to the edge (marked 1st in the plate) and with great ease removed that portion of the bone marked A. I again found the dura mater covered with healthy granulations.

In my second operation, I divided the bone from the edge marked (1st.) to that marked (2d.) and elevated the portion marked B.

In my third operation, I sawed through the remaining bone C. C. C. longitudinally (marked 3d.) and removed the whole of it without using much force.

The dura mater, throughout, was covered with healthy granulations, and the enclosed drawing is a tolerably faithful representation of the appearance of the wound after the removal of the bone.

I found the saw work with great facility, and the operation each time was finished in a few minutes. I think it will be obvious to every one, that had the trephine been used, it would have been laborious and tedious.

The

Mr. Greenhow's Case of Caries of the Os Frontis. 

Printed for Richard Phillips 71 St. Paul's Church Yd. Nov. 1-1804.

The outline of the bone is faithful, and of the exact size.

I have much pleasure in adding, that my patient is progressively getting well.

## Figures and Tables

**Figure f1:**